# Evaluation of skeletal variations and establishment of Cephalometric Norms in Saudi Sub Population using Bjork Jarabak’s analysis

**DOI:** 10.12669/pjms.345.15556

**Published:** 2018

**Authors:** Ibrahim Alshahrani, Muhammad Abdullah Kamran, Ali Alhaizaey, Noura Abumelha

**Affiliations:** 1 Dr. Ibrahim Alshahrani, PhD. Associate Professor, King Khalid University, Abha, Saudi Arabia; 2 Dr. Muhammad Abdullah Kamran, FCPS. Assistant Professor, King Khalid University, Abha, Saudi Arabia; 3 Dr. Ali Alhaizaey, SBO. Assistant Professor, King Khalid University, Abha, Saudi Arabia; 4 Dr. Noorah Abumelha, BDS. Demonstrator, King Khalid University, Abha, Saudi Arabia

**Keywords:** Face, Cephalometry, Population, Male, Female

## Abstract

**Objective:**

To evaluate the skeletal variations amongst individuals and to compare the measurements with the standardized linear and angular values of Bjork Jarabak’s analysis.

**Methods:**

This study was conducted at POS Department, King Khalid University on 100 adult Saudi patients recruited through convenience sampling. It was conducted between April to September 2017, had inclusion criteria of patients between the age group 17 to 22 years showing normal occlusion. After history and examinations, lateral cephalographs were taken, scanned and traced using Dolphin Imaging Software and Cephalometric points were recognized. Linear and angular dimensions were calculated according to Bjork-Jarabak’s method.

**Results:**

Analysis and assessment of Saudi male and female values revealed considerable variation in the anterior and posterior cranial base lengths (p<0.05), anterior and posterior face height, ramus height, and mandibular length. Male measurements in contrast to Jarabak’s values showed noteworthy variances in articular angle, anterior and posterior cranial base, ramus height, length of mandible, anterior face height and Jarabak’s ratio. Female dimensions in relation to Jarabak’s norms showed considerable variances in articular angle, anterior cranial base, posterior facial height with less significant values in Saudi females while compared with Jarabak’s norms apart from mandibular body length which is more in Saudi females.

**Conclusion:**

Skeletal variations amongst Saudi males and females were significant and comparison with standardized linear and angular values of Bjork Jarabak’s analysis was also significant.

## INTRODUCTION

Cephalometric radiography was introduced by Broadbent and Hofrath in 1931 and since then it is widely used as an essential tool in clinical orthodontics for the study of malocclusion and skeletal structure and research orthodontics by researchers.^1^ A cephalometric radiograph and cephalometric norms plays a significant role in assessing the anteroposterior jaw relation^2^, class of occlusion^3^, growth prediction^4^ and act as a substantive tool in Orthodontics which aids Orthodontic clinicians and research workers in the formulation of final treatment plan.^5^

An ethnic group is a nature or populations that shares the same geographic boundary, language or culture and are either historically or racially related.^5^ Diverse ethnic and cultural collection of people have dissimilar facial and dental distinctiveness that justify the role of cephalometric standards for every racial group. Majority cephalometric studies have demonstrated that the ’norms’ should be based on ethnic, sex and age differences.^6^ Studies revealed that the Saudi people has discrete facial and dental appearance when compared with European and American people, as established by diverse analysis.^7^

Arne Bjork practiced dentistry from 1937 to 1951. He gave seven structural signs to determine the mandibular growth rotations. He constructed a facial diagram to determine the distribution of facial pragmatism using the linear and angular measurements. The plane of reference used by him was the SN plane.^8^ Jarabak’s cephalometric investigation was based on the foundation of the exploratory research of Bjork. A facial diagram (polygon) consisting of linear and angular configurations determining the extent of facial prognathous, makes up the Bjork Jarabak’sAnalysis.^4^ One of the basic troubles in orthodontic management is prediction of growth. particularly, Jarabak effort with fragment of the dentofacial complex to judge the association of these section and how they amplify the standard development of any person.

Some studies done previously on Saudi population comprises of small sample size and they have suggested that prospective cephalometric researches on Saudi population from diverse areas of the Saudi kingdom are desirable to confirm the result offered in their study with better sample amount.^9^,^10^ Therefore, this study was planned to analyze the craniofacial pattern of Saudi adults cephalometrically, to evaluate the skeletal variations amongst individuals and to compare the measurements with the standardized linear and angular values of Bjork Jarabak’s analysis.

## METHODS

This cross-sectional study involved the collection and analysis of lateral cephalometric radiographs done at the department of pediatric and orthodontics, King Khalid University and was conducted on 100 non-growing Saudi patients including 50 males and 50 females. The duration of the study was 6 months from April to September 2017. A sample size of 100 patients was recruited through Raosoft software, in which margin of error was 5%, confidence interval of 95%, population size of 134 and response distribution of 50%.

**Fig.1:**
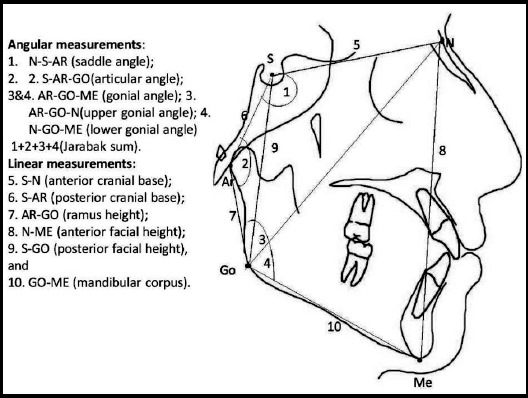
Cephalometric measurements of Jarabak’s analysis.

The inclusion criterion was patients who consented for the study having age group 17 to 22 years old having normal occlusions as based on the British standards institute (Williams and Stephens, 1992). A normal occlusion fulfill the requirements of aesthetics and of function with class I incisor relationship (lower incisor edges occlude with or lie immediately below the cingulum plateau of upper central incisors), subjects that had no previous orthodontic treatment, little or no incisor crowding, no skeletal abnormality, full dentition from second molar to second molar, No craniofacial malformation or syndromes, no history of trauma and whose both parents and grandparents are Saudis. The exclusion criterion was patients who did not gave consent for the study. All the patients were recruited through convenience sampling. After taking history, informed verbal consent and examination, the patients were referred to the radiology department for lateral cephalography and orthopantomography. Every lateral cephalometric radiograph was captured under standardized conditions that is occluded teeth, lips in rest position and head in natural head posture by an experienced radiographer. Every radiograph was scanned into a digital system by means of Epson perfection 4990 photo scanner (Seiko Epson Corporation, Nagano, Japan). The digital tracing was carried out using Dolphin Imaging Software Version 11 (Dolphin Imaging and Management, Chatsworth, CA). The computer analysis software was attuned for the intensification issue by a calibration procedure which includes recognition of identified distance linking two points of the Dolphin ruler on the tracing monitor. Cephalometric markers were identified (Fig.1 and Table-I). Linear and angular measurements were measured according to the Bjork-Jarabak’s Method (Bjork1947, Jarabak *et al*. 1972) (Fig.1) and the inaccuracy/ intra examiner trustworthiness was resolute by taking haphazardly selected 25 radiographs at one-month interval, devoid of references to previous tracings. All measurements were recorded on a predesigned proforma.

**Table-I:** Showing cephalometric measurements of jarabak’s analysis.

Measurement	Definition
***Angular measurements (°)***	
1. Saddle angle: N-S-AR	Measured at the angle between anterior and posterior cranial base.
2. Articular angle: S-AR-GO	Measured at the angle between posterior cranial base and ramus height.
3. Gonial angle: AR-GO-ME	Measured at the angle between ramus height and mandibular plane.
4. Upper gonial angle: AR-GO-N	Measured at the angle between ramus height and Gonion constructed-Nasion line
5. Lower gonial angle: N-GO-ME	Measured at the angle between Gonion constructed-Nasion line and mandibular plane
6. Jarabak Sum	Sum of angles (Saddle angle + Articular angle + Gonial angle)
***Linear measurements(mm)***	
1. Anterior cranial base: S-N	A linear distance from Sella to Nasion.
2. Posterior cranial base: S-AR	A linear distance from Sella to Articulare.
3. Ramus height: AR-GO	A linear distance from Articulare to Gonion constructed.
4. Anterior facial height: N-ME	A linear distance from Nasion to Menton.
5. Posterior facial height S –GO:	A linear distance from Sella to Gonion constructed.
6. Mandibular body length. GO-ME	A linear distance from Gonion to Menton
Proportional measurements (%) % Jarabak Facial Proportion:	A ratio of the Posterior and Anterior facial height

### Control of Error

To control the error in tracings and measurements Dalhberg’s formula (sprigate, 2012) was applied. ME = s (x1-x2)2/2nWhere x1 = the first measurement, x2 = the second measurement, and n = number of repeated records (Houston WJB, 1983) Twenty-five lateral cephalometric radiographs were randomly selected traced and measured at 1-month interval to determine the difference between two readings.

### Statistical Analysis

All the data was recorded and analyzed statistically using SPSS 20 with confidence level set at 5% (P < 0.05). Descriptive analysis including mean and standard deviations were obtained. Independent t test was applied to compare the difference among both the sexes and the difference between Pakistani adults and standard Bjork Jarabak’s value.

## RESULTS

The results of test of level of error revealed that the combined errors for any of the variables were small (less than 0.24 mm for linear measurements and 0.36 degree for angular measurements) and were within acceptable limit.

Descriptive statistics (Means and standard deviation) of craniofacial morphology of Saudi adults are shown in Fig.3. Evaluation of gender difference among Saudis revealed considerable variation in the anterior and posterior cranial base lengths whereas males have superior anterior and posterior face height, ramus height, and mandibular length (p<0.05) (Table-II). The means and standard deviations of angular and linear measurements for Saudi males and females contrast with Jarabak’s norms are presented in Table-III and Table IV. On comparison of cephalometric dimensions of males as contrast to Jarabak’s norms exposed noteworthy dissimilarity in the articular angle, Anterior cranial base, posterior cranial base, ramus height, mandibular length, Anterior facial height and Jarabak’s ratio with greater values in Saudi males as compared to Jarabak’s norms (Table-III). Comparison of findings of females as compared to Jarabak’s norms exposed remarkable differences in the articular angle, anterior cranial base, posterior facial height and mandibular body length. All values were lesser in Saudi females when compared with Jarabak’s norms except mandibular body length which is more in Saudi females (Table-IV).

**Fig.2:**
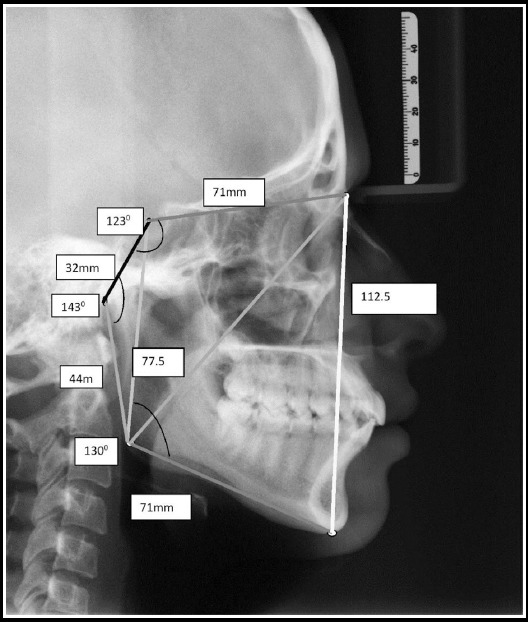
Lateral Cephalograph showing Jarabak norms for Linear and Angular Measurements.

**Fig. 3:**
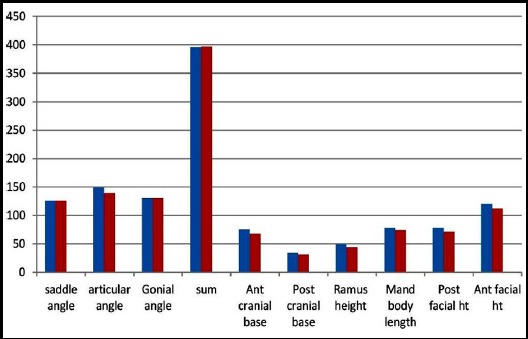
Showing mean of gender distribution of Jarabak norms in Saudi adults.

**Table-II:** Showing statistical significance of different variables of Jarabak Analysis.

Variable	p-value (<0.05)
Saddle angle	0.8724
Articular angle	0.7391
Gonial angle	0.8373
Total sum	0.1358
Ant cranial base	*0.0001
Post cranial base	*0.0025
Ramus height	*0.0001
Mandibular body length	*0.0100
Post. facial height	*0.0001
Ant. facial height	*0.0001
Jarabak ratio	0.988

**Table-III:** Statistical difference between Jarabak’s norms and Saudi males mean.

Variable	Jarabak norms Mean ±SD	Saudi males Mean ± SD	P value
Saddle Angle	123.0 ± 6	125.650 ± 5.163	0.8724
Articular angle	143.0 ± 5	139.806 ± 6.302	0.0076
Gonial Angle	130.0± 6	130.412 ± 6.413	0.5217
Total Sum	396.0 ± 5	395.932 ± 4.604	0.9920
Posterior Cranial Base	32mm ± 3	34.18 ±5.591	0.0162
Anterior Cranial Base	71 mm ± 3	74.644 ±4.851	0.0001
Ramus height	44mm ± 5	49.340 ±4.797	0.0001
Mandibular body length	71mm ± 5	78.327 ± 6626	0.0001
Posterior facial height	77.5 ± 7.5	78.448 ± 7.090	0.3066
Anterior facial height	112.5± 7.5	120.608 ± 6.009	0.0001
Jarabak ratio	63.51 ±1.5	65.04 ± 3	0.0016

**Table-IV:** Statistical difference between Jarabak’s norms and Saudi Females mean.

Variable	Jarabak norms Mean ± SD	Saudi females Mean ± SD	P value
Saddle Angle	123 ± 6	125.842 ± 6.661	0.7337
Articular angle	143 ± 5	139.328 ± 7.916	0.0066
Gonial Angle	130 ± 6	130.182 ± 4.134	0.9802
Total Sum	396 ± 5	397.302 ± 4.504	0.1744
Posterior Cranial Base	32 ± 3	31.11 ± 4.241	0.2286
Anterior Cranial Base	71 ± 3	68.08 ± 4.851	0.0001
Ramus height	44 ± 5	44.250 ± 5.597	0.8143
Mandibular body length	71 ± 5	74.970 ± 6.135	0.0006
Posterior facial height	77.5 ± 7.5	72.020 ± 6.192	0.0001
Anterior facial height	112.5 ± 7.5	112.118 ± 6.099	0.7805
Jarabak ratio	63.5 ± 1.5	64.28 ± 3	0.2944

## DISCUSSION

The results of this study revealed significant difference in most of the variables. Many aspects for example sex, age, ethnic origin, as well as face type, add to facial differences. Overlay on these aspects are individuality which is specific to every person. Since such innate differences are different for every population, values developed for any population should be used only as orientation line and not as complete standards to which all the persons in that population should match and be labeled as “normal”.^11^

Perception of an appealing face is basically subjective by means of ethnicity, age, gender, culture, and personality influencing average facial traits^12^ and different races and ethnic groups have different dental facial pattern thus making cephalometric norms specific to particular ethnic group.^13^ Earlier cephalometric researches performed on Saudi population with reasonable contour and standard occlusion revealed that Saudis have extra protrusive dental and facial distinctiveness in contrast to North Americans and British Caucasian persons.^14^,^15^

Al-Jasser^16^ performed two researches which compares Saudi students and North Americans Caucasians in accordance with Steiner’s and Down’s analyses. It was revealed that Saudi population have different dental and facial characteristics.

Al Barakati and Talic^17^ recognized cephalometric norms in accordance with McNamara’s investigations and evaluate their statistics to values of North Americans and Europeans. It was accomplished that Saudis had class II facial profile as compare to North Americans and Europeans. In additon Saudis have additional protrusive dental and alveolar configuration.

This research suggests that there was only significant increase among the males in the linear measurements in comparison to females whereas angular measurements didn’t show any difference between the two genders which is supported by another study in Saudi population by Nabeel F Talic And Sahar F. Al-Barakati^18^ and Wei^19^ and Foo^20^ who found that in Chinese and Malaysian population respectively, most of the linear measurements were significantly greater in males in comparison to females however in Bangladeshi population there were more significant difference among angular measurements between both the sexes.

Bjork stated that during growth Anterior Facial Height (AFH) should be approximately 2.3 mm/year and Posterior Facial Height (PFH) should2.9 mm/year i.e posterior growth slightly greater than anterior growth. Typical growth of TMJ is anticlockwise as the glenoid fossa and condylar growth surpass anterior vertical growth, thus pushing the symphysis forward. In brachy facial individuals this imbalance is greater and clockwise growth is characteristic of dolichofacial subjects.^14^

Outcome of the present research revealed that Saudi males have considerably more anterior face height in comparison with Jarabak’s values. Judgment of anterior face height is imperative while taking into consideration hyper divergent profiles preceding to treatment planning of orthodontic and/or orthognathic treatments. Comparable outcomes be established by AlBarakati and Talic, which notified the prototype of rearward and downward rotation of the mandible and propensity near a class II prototype amongst the Saudi population.^17^ This can elucidate greater anterior face height detected in current study

Saudi adults PFH/AFH ratio is on the higher side of Jarabak norms for Caucasians resulting in slightly more increase in AFH and downward and backward growth of symphysis. Which is opposite in Pakistani population where PFH/AFH ratio is slightly greater than Caucasians resulting in slightly more increase in PFH and greater advancement of symphysis.^20^

A study carried out on Japanese-Brazilian descendants it was reported that they also had greater PFH/AFH ratio in comparison to Caucasians.^21^

Nada Mahdi Hussein Alhussiny^22^ in Iraqi population found that most of the linear measurements according to Jarabak analysis are greater in Iraqi males compared to Iraqi females and Caucasians which is also supported by our study. At the same time Iraqi females showed greater length of the mandible, posterior cranial base length and articular angle in comparison with Iraqi males whereas our study reported that Saudi Females have lesser above mentioned values than Saudi males.

Abdullah M.Aldrees^23^ in his meta-analysis on lateral cephalometric norms found that Saudis have a greater tendency towards Class II facial pattern and more convex profile than Caucasians which is in favor of our study results.

Dr. Eman I Salama, et al^24^ in their study on Sudan population reported that there was noteworthy sexual characteristics difference in upper and lower anterior facial heights. There is increased upper and lower anterior facial height in males as compared to females which is like Egyptians and Saudis as confirmed by our study.

Mona A Abbassy and Amal Abushal^25^ in their study reported that Saudi females tended to have a more vertical mandibular growth pattern than Japanese which is supported by our study. It has been evaluated from the results of the study and it substantiate the idea that Saudi males and females have distinct cranial and facial appearance when compared to Caucasians. The use of cervical vertebral maturation along with other biological assessment measures can also help to predict the pattern of mandibular growth.^26^ These separate facial appearances should be kept in mind when orthodontists and orthognathic surgeons lay down their management rationales.

It is recommended that further longitudinal multicenter studies with large sample size should be planned in order to bring the results more representative of the population. Orthodontists should consider the specific features of Saudi population while planning their treatment.

### Limitations of the study

It has small sample size and cross sectional design.

## CONCLUSION

The skeletal variations i.e. anterior and posterior cranial base lengths, anterior and posterior face height, ramus height, mandibular length amongst Saudi males and females were significant and comparison with the standardized linear and angular values of Bjork Jarabak’s analysis was also significant.

### Authors’ Contribution

**IAS:** Conceived, writing, critical revision & final Approval.

**MAK:** Data collection, designed, Manuscript writing.

**AAH:** Data analysis, interpretation, writing.

**NA:** Data collection, analysis and interpretation.
